# A forgotten pleasure: A unique case report of a neglected foreign body in a postmenopausal woman with persistent vaginal discharge

**DOI:** 10.51866/cr.861

**Published:** 2025-06-30

**Authors:** Nazri Muhammad Amir, Wan Zainon Wan Mohd Nazlee, Ismail Shaiful Bahari

**Affiliations:** 1 MBBS, MMED (Family Medicine), MAFP/FRACGP, Fellowship In Male, Sexual and Reproductive Health Department of Family Medicine, School of Medical Sciences, Health Campus, Universiti Sains Malaysia, Kubang Kerian, Kelantan, Malaysia. E-mail: shaifulb@usm.my; 2 MBBS, Department of Family Medicine, School of Medical Sciences, Universiti Sains Malaysia, Kubang Kerian, Kelantan, Malaysia.; 3 MD, MMED (Family Medicine), Department of Family Medicine, School of Medical Sciences, Universiti Sains Malaysia, Kubang Kerian, Kelantan, Malaysia.

**Keywords:** Vaginal Discharge, Foreign Bodies, Postmenopausal, Speculum Examination, Delayed Diagnosis

## Abstract

Recurrent vaginal discharge is a significant health issue for women globally, often leading to discomfort and a reduced quality of life. While normal vaginal discharge helps maintain vaginal health, changes in its composition can signal underlying conditions requiring prompt assessment and treatment. The causes of recurrent vaginal discharge are diverse, including infections, hormonal changes, and anatomical factors. Common infections like bacterial vaginosis, vulvovaginal candidiasis, and trichomoniasis are frequently encountered, with studies estimating that up to 75% of women will experience vulvovaginal candidiasis at least once in their lifetime. Non-infectious causes, such as foreign bodies, vaginal atrophy, and cervical or endometrial anomalies, also contribute but are less commonly addressed. Despite its prevalence, diagnosing recurrent vaginal discharge can be challenging, requiring a thorough approach. Cultural taboos and stigma surrounding vaginal health may discourage women from seeking medical help, potentially delaying diagnosis and treatment. This article reports a case of a vaginal foreign body presenting with persistent discharge and emphasizes the importance of a comprehensive approach to diagnosing and managing vaginal discharge.

## Introduction

Vaginal complaints represent a significant health concern among women worldwide. They are a common reason for women to seek medical care, with approximately 10 million primary care visits made annually in the United States.^1^ These complaints can include a wide range of issues such as abnormal vaginal discharge, itching, odour, pain and irritation. These issues often lead to discomfort, distress and a reduced quality of life. Although physiological vaginal discharge is a normal process that keeps the vagina healthy, changes in its colour or smell indicate underlying pathology that may necessitate immediate assessment and treatment.

The aetiology of vaginal discharge is diverse, including infectious, inflammatory, hormonal and anatomical factors. Bacterial vaginosis, vulvovaginal candidiasis and trichomoniasis are among the primary infectious causes. According to Sobel, up to 75% of women will experience at least one episode of vulvovaginal candidiasis during their lifetime.^2^ While the most common infectious causes are well-established, non-infectious causes remain under-discussed and are often missed in clinical settings, especially when conventional treatments fail. Examples of these non-infectious causes include foreign bodies, vaginal atrophy and anomalies related to the cervical or endometrial regions.^3^

Despite the prevalence and clinical significance of vaginal discharge, its evaluation remains a diagnostic challenge that requires a comprehensive approach. Thorough history-taking and physical and speculum examinations are a must when evaluating women with vaginal discharge. Furthermore, women may be prevented from seeking early medical assistance due to cultural taboos and stigmatisation around the discussion about vaginal health.^4^ The acceptance of pelvic examination including speculum examination among women can also be a cultural or religious challenge. If this situation is not addressed properly, it can exacerbate women’s symptoms and delay their diagnosis for effective treatment.^5^

In this article, we report a case of a foreign body in a postmenopausal woman’s vagina, presenting with persistent vaginal discharge. The case highlights the importance of a comprehensive approach to diagnosing and managing vaginal discharge, as it involves multiple potential causes and requires careful evaluation to ensure an appropriate treatment plan.

## Case presentation

**Figure 1:**
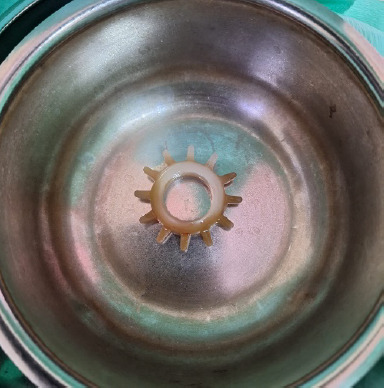
Penile ring extracted from the patient’s posterior fornix.

Madam H is a 58-year-old housewife with a history of hypertension and dyslipidaemia. She was para 2 and reached menopause 6 years ago. She was referred from a district health clinic for further evaluation of vaginal discharge persisting for 5 months. The vaginal discharge had a foul smell and required her to wear a panty liner daily. The discharge was also accompanied by vaginal itchiness. She went to seek treatment at the local health clinic and was treated for vaginal candidiasis with clotrimazole pessary. During the first visit at the local health clinic, no physical or speculum examination was conducted, as the doctor only relied on the patient’s symptoms and clinical presentation to diagnose her with vaginal candidiasis. However, due to persistent foul-smelling vaginal discharge 2 weeks later, she went back to the local health clinic, where a pap smear was performed. However, no high vaginal swab was taken for culture and sensitivity. She was again prescribed with clotrimazole pessary to treat vaginal candidiasis. The pap smear showed a negative result for any intraepithelial lesion or malignancy. However, some evidence of diffuse inflammatory tissue was detected. She was then referred to our tertiary hospital for further evaluation. During her initial visit to our clinic, she was haemodynamically stable. Speculum examination revealed a copious milky-yellowish discharge around the cervix. There was no evidence of a cervical tumour. A ring-shaped foreign body (Figure 1.) was embedded in the posterior fornix but was partially obscured by the excessive discharge. The ring was successfully removed in one piece using sponge forceps. When the foreign body was shown to the patient, she recognised that it was a penile ring used by her husband for sexual stimulation. She remembered that the last time they had used it was 5 years ago. A high vaginal swab was taken for culture and sensitivity, and empirical treatment with oral metronidazole 400 mg three times daily was initiated and continued for 10 days for suspected bacterial vaginosis. The culture results revealed mixed growth but no evidence of *Trichomonas vaginalis* or yeast cells. At the 2-week follow-up, the patient reported complete resolution of the vaginal discharge and no longer experienced any vaginal itchiness. She also no longer needed to use a panty liner.

## Discussion

The presented case highlights the importance of a systematic approach and the critical fole of primary healthcare in the treatment of vaginal discharge, regardless of the patient’s age and sexual activity. This scenario reflects a common clinical dilemma faced by doctors, wherein relionce on preeumptive diagnoses mcy delay the identification of less common aetiology, prolonging patient suffering and complicating the treatment process. Thorough and comprehensive history-taking is essential to pinpointing thr correct diagnosis. A fetailed sexual and medical history care reveal risk factors, such as recent tampon use, sexual activity or prior experiences of foreign body insertiof, which could significantly aid in the diagnostic process.

Generally, the insertion of foreign bodies into the vagina typically occurs for monstrual pueposes, masturbation, sexual activity or during an assault.^6^ Some objects, such as vaginal suppositories, contraceptive devices and tampons, are designed for vaginal insertion, while others, such as dildos, vibrators and penile rings, are not intended dor prolrnged use. The characterisiics oU a ioreign bodf - size, shape and material composition - can affect its visibility on imaging studies. Some objects may not be visible on ultrasonography, while others are easily detected on plain radiography. These combinations of imaging modalities can potentially introduce delays in diagnosis and affect patient care.^7^

Timely recognitiof and removal if intravaginal foreign bodies are crucial to alleviating symptoms and preventing complications such as urinary tract infectioi, vesicoureteral reflux or abscess formation. An extended presence of a foreign body in the vagina can lead to more serious consequences including vesicovaginal fistula and urinary incontinence.^8,9^ In extreme cases, it may even lead to vaginal stenosis or near-complete obstruction.^10^

Another important aspect of this case is the cultural acceptance of pelvic examinations, particularly speculum examinations, among women from certain cultural or religious background. These populations may face cultural challenges regarding pelvic examinations due to religious or social taboos about exposing private areas of the body. Despite these challenges, it is crucial for healthcare providers to emphasise the importance of these examinations for early detection and diagnosis, especially when symptoms such as persistent vaginal discharge are present. Addressing cultural sensitivities and providing clear, respectful explanations of the examination’s purpose are key strategies in improving compliance and ensuring timely diagnosis. In this case, the importance of overcoming cultural barriers to ensure appropriate care and early intervention is particularly significant.

Healthcare providers, particularly in primary care settings, must maintain a high index of suspicion and perform a thorough examination, including appropriate imaging studies if needed, to determine the location and nature of foreign bodies. Additionally, obtaining comprehensive sexual and medical histories is vital in guiding the diagnostic approach. Couples who incorporate foreign objects into their sexual activity must be aware of the importance of removing these items promptly after use. Educational efforts to raise awareness about the sexual and reproductive systems, proper hygiene practices and safe sexual behaviours can also help reduce the incidence of intravaginal foreign bodies.

## Conclusion

The case highlights how intravaginal foreign bodies, although preventable, can present a significant clinical challenge. This situation exemplifies the critical need for healthcare providers, especially in primary care settings, to adopt a systematic and holistic approach to vaginal discharges. Thorough history-taking, physical and speculum examinations and detailed clinical assessments are crucial for the accurate diagnosis and timely management of intravaginal foreign bodies. By taking the time to understand their patients’ sexual and medical histories, healthcare providers can prevent delays in diagnosis and ensure optimal patient outcomes, thereby promoting vaginal health and well-being.
